# THE PITTSBURGH FATIGABILITY SCALE: VALIDATION OF THE MENTAL SUBSCALE IN THE LONG LIFE FAMILY STUDY

**DOI:** 10.1093/geroni/igz038.864

**Published:** 2019-11-08

**Authors:** Sharon W Renner, Patrick J Brown, Todd M Bear, Stacy L Andersen, Stephanie Cosentino, Robert M Boudreau, Adam J Santanasto, Nancy W Glynn

**Affiliations:** 1 University of Pittsburgh Graduate School of Public Health, Pittsburgh, Pennsylvania, United States; 2 Department of Clinical Psychology in Psychiatry, College of Physicians & Surgeons, Columbia University, New York, New York, United States; 3 Department of Behavioral and Community Health Sciences, University of Pittsburgh Graduate School of Public Health, Pittsburgh, Pennsylvania, United States; 4 Department of Medicine, Boston Medical Center, University School of Medicine, Boston, Massachusetts, United States; 5 Department of Neurology, Columbia University Medical Center, New York, New York, United States; 6 Department of Epidemiology, University of Pittsburgh Graduate School of Public Health, Pittsburgh, Pennsylvania, United States

## Abstract

We previously validated the physical, but not the mental subscale of the Pittsburgh Fatigability Scale (PFS). Thus, we aimed to validate the PFS mental subscale in 1,738 individuals aged ≥60 from the Long Life Family Study (55.5% female, age 74.8±11.1 years, PFS mental score 7.1±10.1, range 0-50). Confirmatory factor analysis with promax rotation showed all 10 items loaded on two factors: social and physical activities (SRMR=0.07, RMSEA=0.13, CFI=0.90). PFS mental score had strong internal consistency (Cronbach’s α=0.90) and demonstrated moderate concurrent and construct validity using Pearson correlations against measures of cognition (Trail Making A (r=0.26) and B (r=0.29) time), gait speed (r=-0.30), and the Center for Epidemiologic Studies Depression Scale (r=0.35), p<0.0001 for all. In conclusion, by accounting for self-pacing inherent in common fatigue questionnaires, the validated PFS mental subscore may be a more sensitive tool to examine perceived mental fatigability as an important contributor to cognitive and physical function.

